# Annealing Optimization of Lithium Cobalt Oxide Thin Film for Use as a Cathode in Lithium-Ion Microbatteries

**DOI:** 10.3390/nano12132188

**Published:** 2022-06-25

**Authors:** Akzhan Bekzhanov, Berik Uzakbaiuly, Aliya Mukanova, Zhumabay Bakenov

**Affiliations:** 1 Department of Chemical and Materials Engineering, School of Engineering and Digital Sciences, Nazarbayev University, Kabanbay Batyr Ave. 53, Nur-Sultan 010000, Kazakhstan; akzhan.bekzhanov@gmail.com (A.B.); aliya.mukanova@nu.edu.kz (A.M.); 2 National Laboratory Astana, Nazarbayev University, Kabanbay Batyr Ave. 53, Nur-Sultan 010000, Kazakhstan; 3 Institute of Batteries LLC, Kabanbay Batyr Ave. 53, Nur-Sultan 010000, Kazakhstan

**Keywords:** LiCoO_2_, lithium-ion microbattery, annealing time, temperature ramp rate, annealing temperature

## Abstract

The microbatteries field is an important direction of energy storage systems, requiring the careful miniaturization of existing materials while maintaining their properties. Over recent decades, LiCoO_2_ has attracted considerable attention as cathode materials for lithium-ion batteries due to its promising electrochemical properties for high-performance batteries. In this work, the thin films of LiCoO_2_ were obtained by radio-frequency magnetron sputtering of the corresponding target. In order to obtain the desired crystal structure, the parameters such as annealing time, temperature, and heating rate were varied and found to influence the rhombohedral phase formation. The electrochemical performances of the prepared thin films were examined as a function of annealing time, temperature, and heating rate. The LiCoO_2_ thin film cathode annealed at 550 °C for 1 h 20 min demonstrated the best cycling performance with a discharge specific capacity of around 135 mAh g^−1^ and volumetric capacity of 50 µAh cm^−2^µm^−1^ with a 77% retention at 0.5 C rate.

## 1. Introduction

An overwhelming number of portable electronics are gaining popularity in our daily life and improving the quality of its routine. These high-tech devices continue to demand increasingly higher performance of the batteries and energy storage materials. Nowadays, high-performance Li-ion batteries (LIBs) are favored in electronic applications such as laptop computers, smartphones, drones, and electric cars where the light weight and small volume of batteries have their significance. A number of works have focused on the synthesis, processing, and/or electrochemical characterization of high-capacity cathode materials. Over the last decade, the integration and remarkable improvement of batteries’ technical specifications such as capacity, operating potential, and cyclability have become key limiting factors in the progress of portable electronics. Although electric vehicles, drones, and other types of electronics have already utilized LIBs successfully, their safety and high reliability still need to be improved [1,2]. In the field of energy storage materials, there are niche microapplications, where the total thickness of the microscale energy storage devices should be smaller than 10 µm (nanoelectronics, medical implantable devices, wireless sensors, etc.). Since the first practical thin-film battery was announced by Hitachi Corporation in 1982, intensive efforts have been made to obtain high-performance electrode and electrolyte materials for thin-film batteries [3].

It is well known that the positive electrode of the battery mainly determines the characteristics of the whole cell. Among various cathode materials, LiCoO_2_ (LCO) is one of the most commercialized in the market of both conventional and microscale LIBs due to its excellent electrochemical properties and feasible manufacturing. The main differences between thin-film materials and conventional ones are, first, in thickness, and second, in the method of preparation. The usual electrodes are obtained by slurry casting, which involves the mixing of an active powder material of a pre-known crystal phase with additional conductive additives and binders. Meanwhile, the methods for cathode thin-film preparation contain the simultaneous processes of deposition and material synthesis. Оn the one hand, any extra components in such a process will interfere with the control of the formation of the desired crystal structure; on the other hand, thin-film electrode materials have the advantage of achieving high energy density by reducing inactive components, as well as the sufficient electronic conductivity for charge transfer due to small thickness. For the synthesis of LiCoO_2_ thin films, various methods have been investigated such as magnetron sputtering, sol–gel synthesis, pulsed laser deposition (PLD), chemical vapor deposition (CVD), and laser ablation [4,5,6,7,8,9,10]; however, the obtaining of the pure crystal phase of the LiCoO_2_ film is still challenging and there is still a lack of experimental guidelines on how to obtain a well-working LiCoO_2_ film. Among all methods, sputtering followed by post-deposition annealing is the most easy, scalable, and applicable technique for the deposition of uniform films [4]. Thus, it is very important to obtain the LiCoO_2_ thin films with the necessary crystal structure in order to utilize all its beneficial properties such as high electronic conductivity and capacity [11].

**Figure 1:**
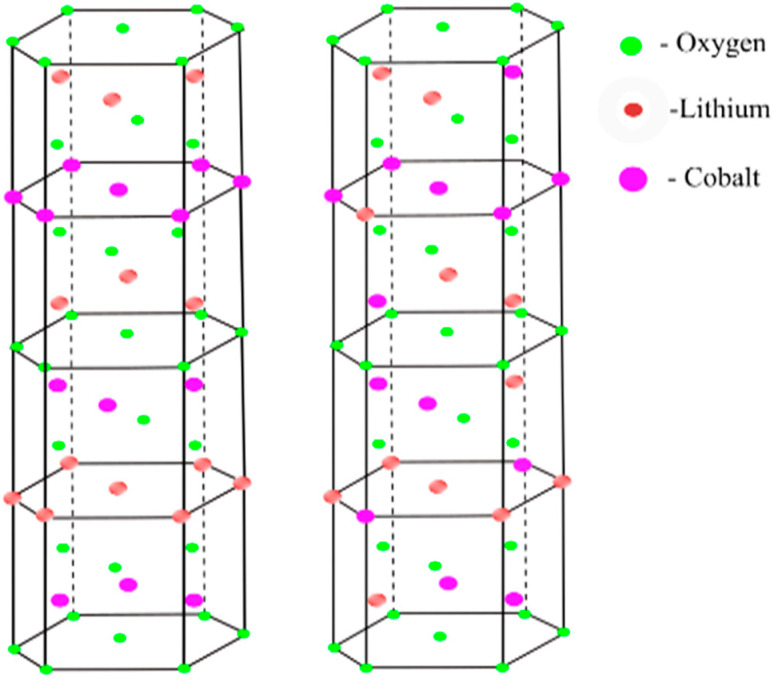
Rhombohedral structure (space group R-3*m*) on the left and cubic structure (space group Fd3m) on the right.

A structural feature of LiCoO_2_ is specified as a hexagonal layered crystal structure (space group R-*3m*), constructed by a cobalt layer and lithium layer alternately occupying octahedral sites between adjacent close-packed planes of oxygen. LiCoO_2_ atoms in crystal (R-3*m*) are in thethe following Wyckoff positions: Co in 3a (0,0,0), Li in 3b (0,0,12), and O in 6c sites (0,0,14) [12]. Two main polymorphic states of the layered oxide compound LiCoO_2_ are known: the rhombohedral phase and the metastable cubic phase, as shown in Figure 1.

A crystallographic structure of a sputtered LiCoO_2_ film was studied under various sputtering conditions, temperatures of annealing, substrates, etc. [13,14,15]. LiCoO_2_ is highly anisotropic; to produce a rhombohedral crystal phase that favors the lithium diffusion at the electrode–electrolyte interface, high-specific-surface-plane-oriented grains are preferred. Intrinsically, there are five structural plane configurations for the rhombohedral structure: (003), (101), (018), (110), and (104). Geometrically, the (003) plane oriented horizontally to the substrate with a low specific surface plane, while the (101) and (104) planes were perpendicular to the substrate with a high specific surface plane [16]. The orientation of the grain can depend on several factors discussed further on. Dudney reported that crystal plane growth depends on film thickness, where 500 nm was found favorable for the (003) plane, and films above 1 µm thick were mostly perpendicular to the substrate orientation [17]. Bates and Yoon showed an influence of the surface energy on crystallographic growth, where it was stated that the (003) plane has less surface energy than (101) and (104), which could be the possible reason for the observed parallel orientation to the substrate [1,18]. Jan et al. investigated LiCoO_2_ films by variations in RF powers (120, 150, and 180 W) and gas ratios (Ar:O_2_, 1:2, 1:1, and 2:1) and found sputtering optimum conditions with an RF power of 180 W and (Ar:O_2_,1:2). A columnar structure with a porous surface morphology was achieved by annealing in air at 700 °C for 1 h at a heating rate of 30 °C min^–1^ [19]. Trusk et al. found the dependence of the formation of the (003) plane from the film thickness and sputtering gas: a gradual increase in thickness above 5 µm and sputtering in a mixture of Ar and O_2_ were found to have an essential effect on the increment in the (003) plane, which is undesirable due to a hindered ionic diffusion [20]. Noh et al. optimized the LiCoO_2_ thin film on an Al substrate by a variation in the working gas ratio (Ar:O_2_) of direct-current (DC) sputtering and post-deposition annealing temperature. During the electrochemical test, a better capacity was observed in the sputtering gas mixture with an Ar:O_2_ ratio of (4:1), though the extent of capacity retention became worse. Regarding the effect of post-deposition annealing temperature, a completely ordered crystal structure has not been obtained [21]. In other works, particularly Kumar et al., Jeon et al., and Pacharova et al., it was justified that the morphological and electrochemical properties, and the elemental ratio of LiCoO_2_ were greatly influenced by sputtering power [4,22,23].

In the present work, being interested in different and sometimes contradictory data discussed above, we continued the experimental studies of LiCoO_2_ thin film deposited by RF-magnetron sputtering. The optimal sputtering conditions were chosen on the basis of earlier published literature data. The post-deposition annealing conditions such as heating rate, annealing duration, and temperature were optimized to obtain the thin films of LiCoO_2_ with a rhombohedral crystal phase. The experiments were accompanied by characterization methods such as scanning electron microscopy (SEM), X-ray diffraction (XRD), X-ray photoelectron spectroscopy (XPS), Atomic Force Microscopy (AFM), cyclic voltammetry (CV), and galvanostatic cycling. The obtained experimental results provided the key understanding of factors mainly influencing the formation of the rhombohedral crystal phase in RF-sputtered LiCoO_2_ thin film.

## 2. Materials and Methods

### 2.1. Materials

Lithium Cobalt Oxide Target (LiCoO_2_; 99.9% purity; 5.08 cm in diameter; density of 4.74 g cm^−^^3^; Kurt J. Lesker, Sussex, UK); monocrystalline SiC thin film (300 nm) on Si wafer (525 µm) (Advanced Epi, Leamington Spa, UK); platinum sputtering target (5.08 cm in diameter; 99.99% purity; Angstrom Engineering, Kitchener, ON, Canada); ethanol; acetone; stainless-steel current collector with a diameter of 1.54 cm.

### 2.2. Sputtering of LiCoO_2_ Thin Films

SiC/Si wafers and stainless-steel substrates were ultrasonically cleaned in an ethanol and acetone mixture at 40 °C for 15 min, then rinsed with distilled water and left to dry in a furnace at 100 °C. A 300 nm thick monolithic Pt film barrier underlayer was deposited on Si/SiC and SS (stainless steel) substrates with a DC sputtering source at a power of 180 W in an Ar atmosphere (Angstrom Engineering magnetron sputtering system).

A 1.2 µm thick LiCoO_2_ thin film was deposited by an RF (13 MHz frequency) source at a power of 90 W in an Ar:O_2_ ratio of (5:1). The optimal sputtering conditions were chosen on the basis of earlier published literature data [1,2,3]. The post-deposition annealing was performed in a tubular furnace and rapid thermal annealing (RTA) equipment. Several heating rates of annealing were examined: 10 °C min^−^^1^, 20 °C min^−^^1^, 30 °C min^−^^1^, “instant” (immersing samples into heated tube furnace where argon was purged), and 1200 °C min^−^^1^ for RTA. However, the samples annealed at the ramp rates of 10 °C min^−^^1^ and 20 °C min^−^^1^ did not show any structural differences; therefore, experimental data concerning the latter were omitted. A mass loading of LiCoO_2_ was estimated by measuring the samples before and after deposition using a high-precision microbalance ().

### 2.3. Electrochemical Characterization

The electrochemical tests were conducted with the successfully obtained crystalline LiCoO_2_ thin film with the use of CR2032 coin-type cells assembled in an Ar filled glovebox (Inert MBRAUN, Munich, Germany, pure argon, O_2_ < 0.1 ppm, and H_2_O < 0.1 ppm). An amount of 1 M LiPF_6_ in EC:DEC:EMC (ethylene carbonate/diethyl carbonate/ethyl methyl carbonate) (1:1, vol%) was used as an electrolyte, a Celgard^®^ 2400 polypropylene film served as a separator, and an Li metal chip acted as an opposite and reference electrode. Electrochemical Impedance Spectroscopy (EIS), cyclic voltammetry (CV), and galvanostatic cycling were performed on the BioLogic, (Seyssinet-Pariset, France (VMP3)) and Neware battery testers.

### 2.4. Material Characterization

X-ray powder diffraction (Rigaku SmartLab^®^, Tokyo, Japan) was used for crystal structure observation. The XRD patterns of the deposited LiCoO_2_ were analyzed by the High Score Plus 2018 licensed version program [24]. An SEM microscope (Crossbeam 540, Zeiss, Oberkochen, Germany) coupled with energy-dispersive X-ray spectroscopy (EDS) was employed to observe the morphology analysis of the samples. The deposition rate and thickness were controlled by a quartz crystal microbalance. The thickness of the films was confirmed by a profilometer (Dektak XT Stylus Profiler by BRUKER, Billerica, MA, USA). A SmartSPM 1000 Scanning Probe Microscope (AFM by AIST-NT, Novato, CA, USA) was used to probe the topology and morphology of the prepared materials. The surface characterization of films was analyzed by a Nexsa X-Ray Photoelectron Spectrometer (XPS by Thermo Fisher Scientific, Waltham, MA, USA).

## 3. Results

### 3.1. X-ray Diffraction Analysis

**Figure 2:**
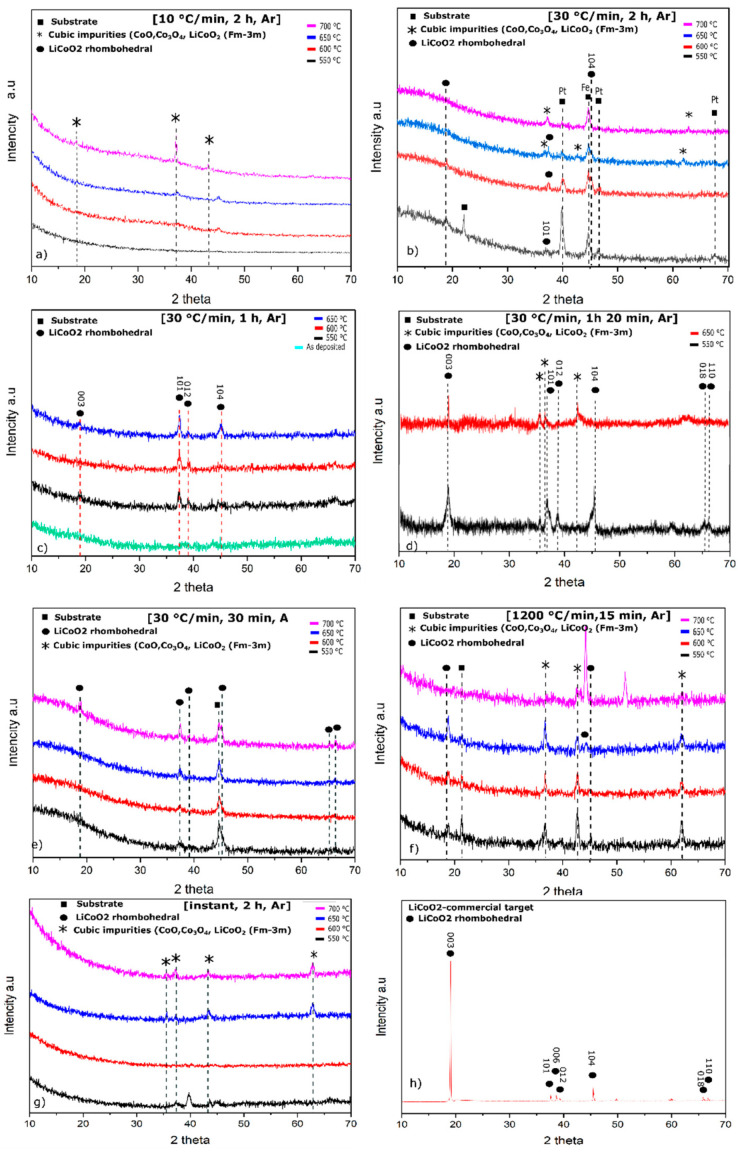
XRD pattern of annealed LiCoO_2_ thin films in Ar atmosphere with changing annealing times, temperatures, and ramp rates: (**a**) 10 °C min^−1^, annealed 2 h; (**b**) 30 °C min^−1^, 2 h; (**c**) 30 °C min^−1^, 1 h; (**d**) 30 °C min^−1^, 1 h 20 min; (**e**) 30 °C min^−1^, 30 min; (**f**) 1200 °C min^−1^, 15 min; (**g**) instant, 2 h; (**h**) commercial target.

In order to identify the crystallinity of the deposited LiCoO_2_ thin films, XRD analysis was utilized. Diffraction patterns of thin films prepared at varying annealing conditions were measured. Figure 2 demonstrates the XRD of LiCoO_2_ samples deposited on SS/Pt substrates and annealed in the temperature range between 550 °C and 700 °C in an Ar atmosphere with the following temperature ramp rates: 10 °C min^−^^1^, 30 °C min^−^^1^, “instant”, and 1200 °C min^−^^1^. The annealing time varied between 0.5 and 2 h for treatment in a tubular furnace and 15 min for RTA, while the cooling time until room temperature was registered to be around 2 h for the tube furnace and 30 min for RTA.

From Figure 2a, the annealing at a 10 °C min^−^^1^ ramp rate did not lead to the formation of any rhombohedral R-3*m* crystalline structure along the range of 550–700 °C, where only the cubic phase impurities, particularly the CoO and Co_3_O_4_ phases, were observed at 700 °C, which is in a contrast with earlier literature data [25,26]. The phase transformation between CoO and Co_3_O_4_ probably occurred due to the structural relationships between the tetrahedral and octahedral oxygen anions upon a reductive change from trivalent to divalent cobalt cations under an argon atmosphere during heat treatment and active lithium evaporation upon long-term heating and ramp rates [27]. Figure 2b shows the XRD patterns of films annealed at a ramp rate of 30 °C min^−^^1^. At 550 °C and 600 °C, the formation of hexagonal phases was identified in the minor phase, and intensity peaks were weak. The observed peaks were interpreted as follows: (003) plane at 19°, (101) plane at 37.4°, and (104) plane at 45.5°, while planes (018) and (110) were not noticed and were probably obscured under substrate noise. Considering the spectra of the samples annealed at 550 and 600 °C, one can notice the (104) plane’s peak at 45.5° that was mainly shaded by the intense substrate peak of SS. The films annealed at 650 and 700 °C, on the contrary, evidence the formation of cubic phases. Overall, the cubic and hexagonal phases coexisted in the latter pattern in Figure 2b. The substrate peaks such as Pt (111) and Fe (111) suppressed upon increasing temperature. The presence of the hexagonal LiCoO_2_ phase is usually determined by a distinct separation of the (110) and (018) peaks or by the appearance of the (006) and (012) peaks in the XRD pattern, which is shown in the target’s reference XRD pattern in Figure 2h for comparison. It was reported earlier [28] that the substrate peak of Pt (111) assists in the formation of a hexagonal phase (104) plane upon long-term annealing at 700 °C. Herein, the preferred orientation (104) plane was not observed (Figure 2c), which can supposedly indicate that the substrate does not affect plane formation.

The films annealed for 1 h with a ramp rate of 30 °C min^−^^1^ (Figure 2c) at 550 °C formed signals of (018) and (110) planes at 65° and 66°, respectively, although both merged into one signal without a noticeable separation, while for the films annealed at 650 °C, the planes (003), (110), (012), and (104) clearly formed, evidencing the R-*3m* phase. For the films annealed at 600 °C, planes (003) and (104) were suppressed, perhaps due to the unfinished phase transition from amorphous to crystalline (recrystallization phenomenon).

The samples annealed for 1 h 20 min (Figure 2d) demonstrated completely formed rhombohedral phase planes, especially the splitting of (018) and (110) at 550 °C. The intensities of peak planes (104) and (003) are comparably equally high. It has also been well known that (003) and (104) peaks are the main ones determining the degree of ion ordering in LiCoO_2_ powder. Meanwhile, the samples annealed at 650 °C demonstrated the suppression of (101), (012), (104), (018), and (110) peaks and narrowing of the (003) signal, while lithium-deficient cubic-phase signals increased. It was reported [29] that with an increase in the *I*(003)/*I*(104) ratio (“*I*” is the XRD peak intensity), the LiCoO_2_ crystallinity was enhanced, i.e., a well-ordered layered structure formed at 550 °C, at which our prepared crystalline LiCoO_2_ thin films exhibited the preferred orientation and confirmed the abovementioned observation performed by Dahn et al., and Bates et al. [18,29] acknowledged that the films above 1 µm acquired a texture during the annealing process where the majority of the grains were oriented with (101) and (104) planes that formed parallel to the substrate due to the minimization of volume strain energy. Bohne [30], on the contrary, noticed that there was no rule on preferential LiCoO_2_ film orientation, rather the chemical structure of the surface. The results of XRD in Figure 2d showed that the film thickness and surface chemical structure were found as major effects for crystal plane orientation. Thus, the step increase by 100 °C between two XRD patterns induced the recrystallization from the initial hexagonal to cubic, more likely due to lithium loss or migration.

In Figure 2e, the annealing time was shortened up to 30 min (30 °C min^−^^1^), which favored the formation of a rhombohedral structure only at 700 °C, which can be seen as a splitting of (018) and (110) planes at 65.2° and 66.5°, respectively. Meanwhile, the samples heated at the rest of the temperatures showed only an XRD pattern related to substrate noise. RTA-annealed (1200 °C min^−^^1^) cathode films were mainly enriched by cubic impurities and, in the minority (space group R-*3m*) rhombohedral phase that was detected as (003) and (104) planes in Figure 2f. Instantly annealed samples (Figure 2g) also led to the formation of unfavorable cubic phases at 650 and 700 °C.

Overall, considering the above-demonstrated XRD of thin films, rhombohedral structure formation preferentially occurs at a heating rate of 30 °C min^−^^1^ and annealing for 1 h 20 min at 550 °C in an Ar atmosphere. Some key points can be mentioned in terms of annealing temperature where 700 °C isothermal holding should be less than 30 min, while at 650 °C, around 1 h is required to form a hexagonal crystalline structure.

There are two common mechanisms by which atoms can diffuse through a solid: substitutional—usually diffuse by a vacancy mechanism; interstitial—the smaller interstitial atoms migrate by forcing their way between the larger atoms. The diffusion energy possessed by each atom is given by 3 kT and increases in proportion to the absolute temperature. Lithium atom diffusion or migration according to [31] is governed by a single vacancy mechanism, where Li diffusion increased exponentially with increasing temperature, which obeys the Arrhenius law, Equation (1), where activation enthalpy Δ*H* and a pre-exponential factor *D*_0_:

(1) $$D=D_{0}exp\left(-\frac{∆H}{k_{B}T}\right),$$

where $D_{0}=xa^{2}v_{0}\exp\left(∆S/k_{B}\right)$, *a—*jump distance, *x—*the mole fraction of Li vacancies, *v*_0_—characteristic vibration frequency, ∆*S*—entropy, and *k_B_*—Boltzmann gas constant. 

According to the theoretical assumption in the work [32], Li atoms migration at 250 °C can take place even at the order of tens of nanometers. Rahn et al. [31] observed the in-depth diffusion of Li atoms at an elevated temperature, where their penetration depths at 400 °C during annealing times of 5 min and 30 min were 171 and 351 nm, respectively. At a low heating rate, the diffusion of atoms (Equation (1)) is in delayed manner, resulting high the energy barrier for nucleation; therefore, Li atoms partially tend to migrate to grain boundaries until reaching a sufficient crystallization temperature, at which Li atoms are supposed to participate in the crystal formation. The rapid heating rates (1200 °C min^−^^1^ and instant) associate a high driving force and low-activation-energy barrier, where, according to [33], an initially heterogeneous nucleation rate dominates, and then homogeneous nucleation provides the highest nucleation rates. Therefore, heating rate and timing in crystallization are assumed to influence the crystallization kinetics of thin films, where thermodynamic models are suggested to understand the film-temperature-dependent crystallization.

From the patterns in Figure 2b,f,g, the substrate peaks were depressed with rising temperature, which is assumed primarily due to the substrate surface roughness melting, where melting temperatures in bulk T_m_ and on the surface T_s_ differ; as a result, nanosize particles on the substrate surface start to melt at much lower temperatures than in bulk [34,35,36,37,38,39,40].

**Figure 3:**
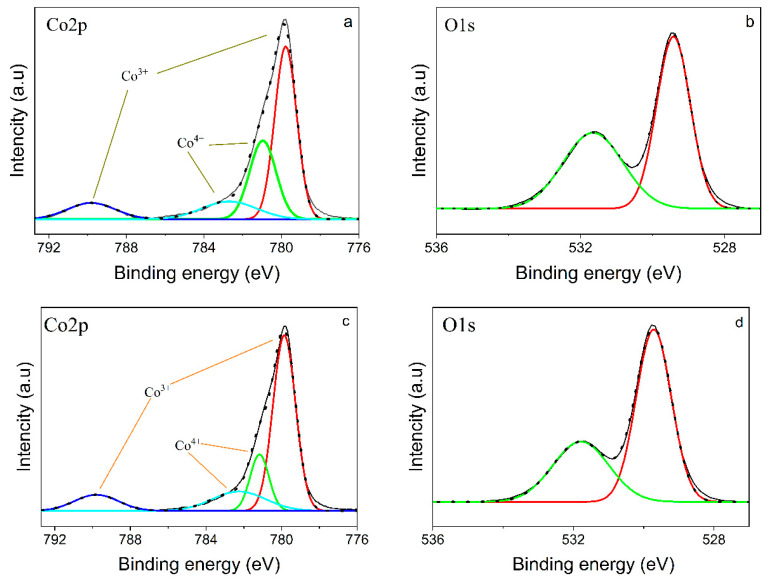
XPS spectrum of Co2p and O1s of thin film annealed (**a**,**b**) at 550 °C for 1 h 20 min in Ar; (**c**,**d**) at 650 °C for 1 h 20 min in Ar.

In order to study the oxidation states of the obtained thin-film samples, XPS was performed, as shown in Figure 3, the results of which revealed that the Co^3+^ and Co^4+^ ions were present on the surface of the oxides films. In Figure 3a,c, the fitted doublet at 779.7 eV and 789.7 eV was associated with Co^3+^; in addition, no shifts were observed, excluding the Co^2+^ presence, according to work [40]. The binding energy peaks at 781.12 eV and satellite 782.6 eV identify the structure characteristic of Co^4+^ ions in the LiCoO_2_ thin films. The main contributor in the crystalline LiCoO_2_ is expected to be Co^3+^ ions. The O1s also allows the oxidation state to be understood. In Figure 3b,d, O^2−^ appeared at 529.7 eV, which is assumed to belong to LiCoO_2_ octahedral sites, whereas at a higher binding state, the O1s is associated with an oxidizing environment [41,42]. The analyses of Li1s showed one peak environment at 54.2 eV corresponding to LiCoO_2_.

### 3.2. Morphological Investigation

**Figure 4:**
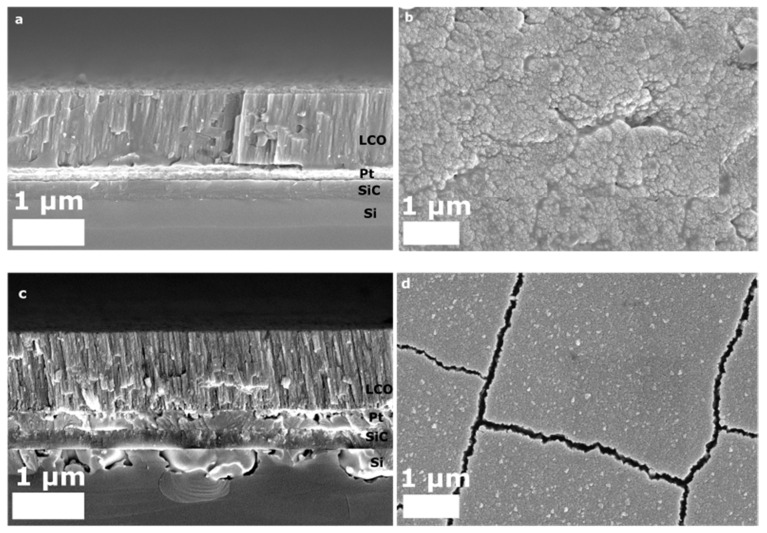

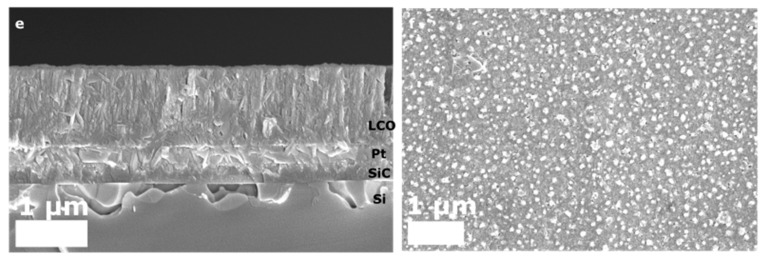
SEM images of LiCoO_2_ thin films on Pt/SiC/Si: (**a**,**b**) as-deposited and nonannealed; (**c**,**d**) cross-sectional and surfaceviewsof film annealed at 550 °C with a ramp rate of 30 °C min^−1^ and annealing time for 1 h 20 min in Ar; (**e**,**f**) cross-sectional and surface morphology of LCO/Pt/SiC/Si at 650 °C with a ramp rate of 30 °C min^−1^ and annealed for 1 h 20 min in Ar atmosphere.

The samples of 1.2 µm thick LiCoO_2_ films on a 300 nm thick Pt layer were investigated by SEM in order to check the cross-sectional and surface morphology (Figure 4). The Pt interlayer acted as an anti-diffusion barrier and current collector layer on the SiC/Si substrate. The cross-sectional image and top surface view demonstrate a uniform deposition, which can be observed in Figure 4a,b. Figure 4c illustrates the cross-sectional image of samples annealed at 550 °C for 1 h 20 min at a ramp rate of 30 °C min^−^^1^. The film structure shows the columnar structure throughout the cathode film, which is in agreement with the formation of a perpendicular-to-substrate, (104)- and (101)-plane-oriented structure [43]. In Figure 4d, the top-view image shows the formation of vivid grains with boundaries on the surface [1,18]. From Figure 4d,e, the formation of cracks throughout the layer of the cathode can be obviously seen, which was more likely caused by the stress release as a result of the difference in thermal expansions of the substrate and cathode. On the other hand, the presence of cracks and the formation of columnar structures facilitated an ionic diffusion by forming a higher surface area and improving the battery cycle life. In Figure 4e, the morphology of the film upon long-term heating at 650 °C was observed to shrink and become more dispersed. The surface image in Figure 4f indicates that grain boundaries were not pronounced and columns were not formed, and the films structure across the film became rougher and without formed grains. This is an indicative signal of a prevailing cubic phase across the film structure, which does not contradict the XRD pattern of the sample in Figure 2d.

**Figure 5:**
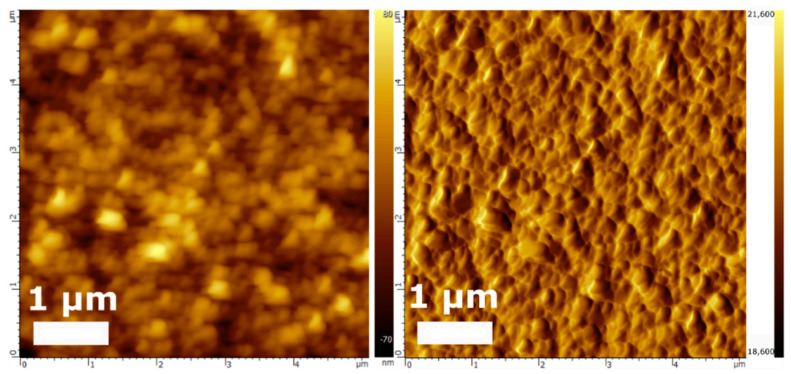
AFM images of LiCoO_2_ thin film annealed at 550 °C for 1 h 20 min in Ar atmosphere.

Figure 5 shows the AFM surface images on the projected area (5 µm × 5 µm) of the annealed LiCoO_2_ film corresponding to those demonstrated in Figure 4c,d. The scanned topology reveals particles on the surface where the average grain roughness radius (R_a_) is calculated to be around 61 nm and the average surface roughness (R_h_) is found to be around 91 nm.

### 3.3. Electrochemical Performance

**Figure 6:**
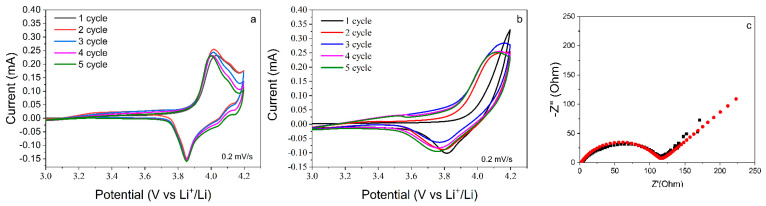
CV scans of LiCoO_2_ thin-film cathodes annealed in Ar: (**a**) 550 °C for 1 h 20 min at ramp rate of 30 °C min^−1^; (**b**) 550 °C annealed for 2 h at 30 °C min^−1^; (**c**) EIS for 550 °C for 1 h 20 min at ramp rate of 30 °C min^-1^ before cycling at discharge state.

After careful characterization with the identification of the crystal structure and morphological features of 1.2 µm thick LiCoO_2_ films, the electrochemical properties were assessed for the most crystalline samples. In order to evaluate Li_x_CoO_2_ formation and major oxidation/reduction peaks, CV was performed in the potential range of 3.0–4.2 V at a scan rate of 0.2 mV s^−^^1^. Figure 6a shows the cathodic peaks at potentials of 4.01 V and 3.85 V associated with the redox peaks corresponding to (0.75 ≥ x ≥ 0.95), where the structure transforms from rhombohedral insulating into rhombohedral metallic [44], while anodic peaks at 4.2/4.15 V and 4.21/4.14 V, according to [45], refer to (0.5 ≥ x ≥ 0.75), which is the phase transition from the rhombohedral to monoclinic structure through the in-plane ordering of Li atoms. In Figure 6b, the peaks at 4.15/3.85 V associated with redox peaks [confirm that the electrochemical ionic diffusion occurred despite the formation of small peaks not being detected.

In order to understand the kinetics properties, EIS scans were measured for the crystalline thin film before cycling, as shown in Figure 6c. A semicircle represents the charge transfer process across the interface between the electrode and the electrolyte. The charge transfer resistance of the LiCoO_2_ cathode film annealed at 550 °C made at near 115 Ohm favored fast Li ion diffusion.

**Figure 7:**
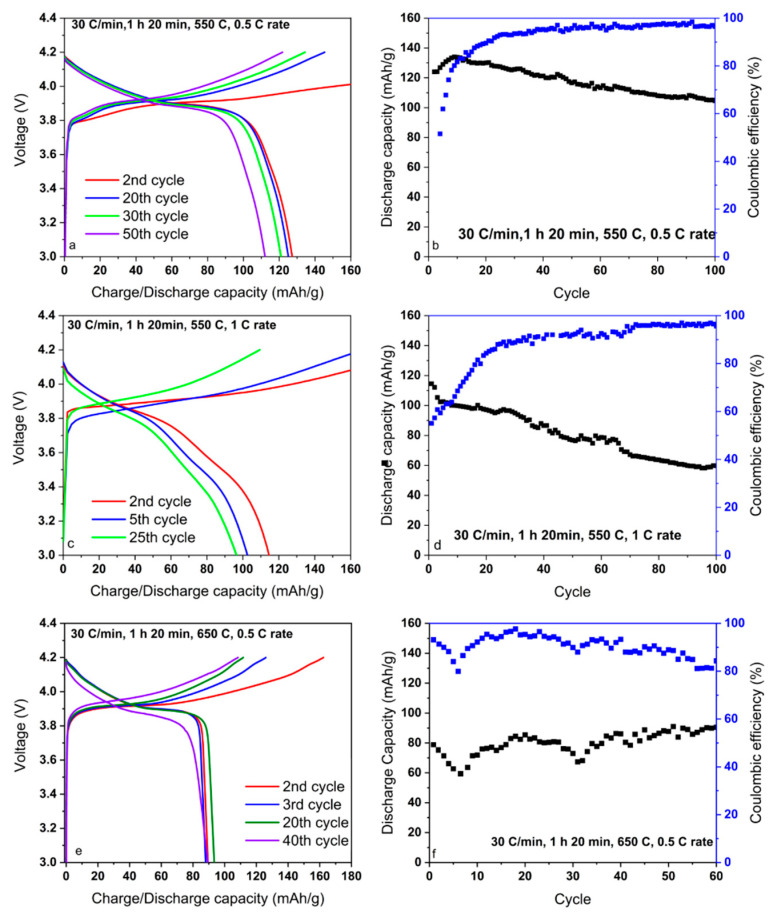
Potential profiles and cycling performances of LiCoO_2_, respectively: (**a**,**b**) 1 h 20 min annealed with ramp rate of 30 °C min^−^^1^ at 550 °C with 2 initial cycles by 0.1 C, hereafter 0.5 C; (**c**,**d**) 1 h 20 min with ramp rate of 30 °C min^−^^1^ at 550 °C at 1 C; (**e**,**f**) 1 h 20 min for 650 C at 0.5 C.

The potential profiles and cycling performance are given in Figure 7. As can be seen in Figure 7a,b, the two initial formation cycles were performed at 0.1 C rate followed by an increase in C rate up to 0.5 and 1 C for further comparison. Hereafter, the cell cycled at 0.5 C tended to gradually increase in discharge capacity during the initial 10 cycles up to 135 mAh g^−^^1^ due to activation of ion diffusion areas. Afterward, the capacity decreased until 105 mAh g^−^^1^ in the 100th cycle, while the Coulombic efficiency (CE) remained above 90% in the second half of the test. At 1 C rate, the CE increased in a delayed manner up to 70 cycles, from 70 to 95% accordingly (Figure 7c,d); meanwhile, the capacity showed a dropping trend up to 55 mAh g^−^^1^. The good capacity retention of the cell is most likely due to the completely formed hexagonal crystal planes (101), (104), and (110) that provide an unhindered intercalation and deintercalation of Li ions. Similarly, the cycling performance of samples annealed at 650 °C for 1 h 20 min was tested. The lattershowed fluctuating capacity behavior around 90 mAh g^−^^1^ and erratically decreasing CE, perhaps in the force of the formation of unfavorable cubic impurities (Figure 7e,f).

For comparison, an existing scholar collected data for LiCoO_2_ thin-film cathodes, as summarized in Table 1, including the deposition conditions, annealing conditions, type of battery, and electrochemical cycling performances. In the majority of works below, authors utilized various approaches such as substrate heating during deposition for in situ crystallization, and laser and RTA patterning for the increase in active areas. Several works lacked exact annealing condition data and did not show the full rhombohedral R-3*m* phase plane formation. In the present work, the route to achieve the pure crystalline LiCoO_2_ thin film at a relatively low annealing temperature without utilizing additional influencing factors was demonstrated. This work provides helpful and effective guidelines for obtaining the LiCoO_2_ thin-film cathode with a discharge specific capacity of around 135 mAh g^−^^1^ and volumetric capacity of 50 µAh cm^−^^2^µm^−^^1^ with a 77% retention at 0.5 C rate. The capacity retention of the obtained cathodes can be improved with the use of a solid electrolyte instead of a liquid one due to the delayed electrochemical reaction and material degradation in the former case. Furthermore, the investigated LiCoO_2_ thin-film cathode can be easily prepared from economically commercially available material that can readily be used for further applications in lithium-ion microbatteries.

## 4. Conclusions

To conclude, LiCoO_2_ thin films deposited through RF magnetron sputtering in a gas ratio Ar:O_2_ of (5:1) and annealed in pure Ar atmosphere were investigated in the temperature range of 550–700 °C, varying the heating rate and annealing time. From the experimental observations, in order to reach the rhombohedral phase (R*-3m* space group) in the deposition Ar:O_2_ (5:1) atmosphere, tweaking the post-deposition annealing temperature, time, heating rate, and annealing atmosphere contributed to the desired phase formation. Following the deductions upon long-term heating rates and annealing time, the Li loss occurred and led to the formation of cubic phase impurities in the film. Conversely, increasing the heating rate and optimizing the annealing time relative to temperatures in the Ar atmosphere effectively contributed to the formation of a hexagonal phase according to XRD, XPS, and electrochemical test results. The effects of heating rate and temperature on the crystallization of LiCoO_2_ thin films were discussed from the viewpoint of thermodynamics and kinetics for a better understanding of the mechanism of the amorphous-to-crystalline phase transition on the level of crystal growth nucleation.

This work provides helpful and effective guidelines for obtaining the LiCoO_2_ thin-film cathode with a discharge specific capacity of around 135 mAh g^−^^1^ and volumetric capacity of 50 µAh cm^−^^2^µm^−^^1^ with a 77% retention at 0.5 C rate. The capacity retention of the obtained cathodes can be improved with the use of a solid electrolyte instead of a liquid one due to the delayed electrochemical reaction and material degradation in the former case. Furthermore, the investigated LiCoO_2_ thin-film cathode can be easily prepared from economically commercially available material that can readily be used in further applications in lithium-ion microbatteries.

## Figures and Tables

**Table 1:** Summarized table with existing scholar-collected data for LiCoO_2_ thin-film cathodes.

#	Material Type	Deposition Condition, (Deposition Gases, Heating Substrates, Power of Sputtering)	Post Deposition Conditions	Thickness	Micro Battery Type	Initial Discharge Capacity	Voltage Range	Current Rate, Retention %	Num. of Cycles	Ref.
1	LiCoO_2_ film	Ar:O_2_ (3:1), heated substrate at 500 °C (in situ annealing)	-	<1 µm	Li/liquid electrolyte/LiCoO_2_	63 µAh cm^−2^ µm^−1^	3–4.2 V	1 C, 84%	100	[46]
2	LiCoO_2_ film	Ar, in situ heated substrate 300 °C and 600 °C	Annealing by RTA 10 min at 600 °C in Ar	0.7 µm	Li/liquid electrolyte/LiCoO_2_ Li/LIPON/LiCoO_2_	25 µAh cm^−2^ µm^−1^ 60 µAh cm^−2^ µm^−1^	3–4.2 V 3–4.2 V	1 C, 85% 5 C, 100%	50 100	[47]
3	LiCoO_2_ film	Ar:O_2_ (3:1) and (5:1), DC power 130 W	Annealed at 500 °C in atmosphere	-	Li/liquid electrolyte/LiCoO_2_	46 µAh cm^−2^ µm^−1^	3–4.2 V	0.1 C, 8.2%	100	[21]
4	LiCoO_2_ film	Ar:O_2_ (96:4%),	Annealed at 800 °C in Air	10 µm	Li/LIPON/LiCoO_2_	60 µAh cm^−2^ µm^−1^	3–4.2V	0.1 C, 95%	100	[20]
5	LiCoO_2_ film	Ar	Annealed at 550 °C, holding time 20 min at O_2_	1.1 µm	Li/liquid electrolyte/LiCoO_2_	37.5 µAh cm^−2^ µm^−1^	3–4.2 V	0.1 C, 3.8%	50	[48]
6	LiCoO_2_ film	Ar:O_2_ (1:2, 1:1, and 2:1), RF power 120, 150, and 180 W	1 h at 700 °C in air	1.6 µm	Li/liquid electrolyte/LiCoO_2_	16.7 µAh cm^−2^ µm^−1^	3–4.2 V	0.2 C	20	[19]
7	LiCoO_2_ film	Ar, laser-patterned	400 °C and 600 °C in Ar:O_2_ (1:5) 3 h	3 µm	Li/liquid electrolyte/LiCoO_2_	140 mAh/g	3–4.2 V	0.05 C, 67%	30	[49]
8	LiCoO_2_ film	Ar:O_2_, in situ substrate heated at 250 °C	In O_2_ two hours 500 °C 600 °C 700 °C	>1 µm	Li/liquid electrolyte/LiCoO_2_	41.8 µAh cm^−2^ µm^−1^ 52.6 µAh cm^−2^ µm^−1^ 61.2 µAh cm^−2^ µm^−1^	3–4.25 V	10 µA cm^−2^, 58%, 72% 74%	50	[28]
9	LiCoO_2_ film	Ar:O_2_ (3:1), different deposition pressure parameters changed	500 °C 2 h in air	<1 µm	Li/liquid electrolyte/LiCoO_2_	67 µAh cm^−2^ µm^−1^	3–4.2 V	0.2 C, 95%	50	[26]
10	Zr doped LiCoO_2_ film	Ar:O_2_ (9:1), in situ substrate heated at 250 °C	600 °C 3 h in air	>1 µm	Li/liquid electrolyte/LiCoO_2_	64 µAh cm^−2^ µm^−1^	3–4.2 V	1 C, 98.5%	25	[50]
11	LiCoO_2_ film	Ar,	400–700 °C in O_2_	<1 µm	Li/LIPON/LiCoO_2_	40 µAh cm^−2^ µm^−1^ (80 mAh g^−1^)	3.3–4.2 V	0.01 C, 78%	5	[51]
12	LiCoO_2_ film	Ar:O_2_ (4:1), DC power 180 W	600 °C in O_2_	0.5 µm		30.7 µAh cm^−2^ (or 56.9 µAh cm^−2^ µm^−1^)	3–4.2 V	10 µA cm^−2^, 76%	30	[52]
13	LiCoO_2_ film	Ar:O_2_ (3:1), RF power 100 W, in situ-heated substrate 400 °C	-	0.4 µm	Li/liquid electrolyte/LiCoO_2_	54.5 µAh cm^−2^ µm^−1^	3–4.2 V	10 µA cm^−2^, 58.2%	50	[53]
14	ZrO_2_ coated LiCoO_2_ film	Ar:O_2_ (4:1), DC power 100 W	600 °C 1 h in O_2_	0.6 µm	Li/liquid electrolyte/LiCoO_2_	12.2 µAh cm^−2^ µm^−1^	3–4.5 V	10 µA cm^−2^, 75%	40	[54]
15	LiCoO_2_ film	Ar:O_2_	300–700 °C 1 h in air	>1 µm	Li/liquid electrolyte/LiCoO_2_	132 mAh g^−1^ (or 62 µAh cm^−2^ µm^−1^)	3–4.3 V	0.1 C, 70 %	50	[55]
16	LiCoO_2_ film	Ar:O_2_ (5:1), RF power 100 W	550 °C, 1 h 20 min annealed in argon	1.2 µm	Li/liquid electrolyte/LiCoO_2_	135 mAh g^−1^ (50 µAh cm^−2^ µm^−1^) 135 mAh g^−1^ (50 µAh cm^−2^ µm^−1^) 115 mAh g^−1^ 42 µAh cm^−2^ µm^−1^	3–4.2 V	0.1 C, 93% 0.5 C, 77% 1 C, 50%	20 100 100	Our data

## Data Availability

The data presented in this study are available on request from the corresponding author.
